# Exploration of muscle loss and metabolic state during prolonged critical illness: Implications for intervention?

**DOI:** 10.1371/journal.pone.0224565

**Published:** 2019-11-14

**Authors:** Liesl Wandrag, Stephen J. Brett, Gary S. Frost, Vasiliki Bountziouka, Mary Hickson

**Affiliations:** 1 Section for Nutrition Research, Department of Metabolism, Digestion and Reproduction, Imperial College London, England, United Kingdom; 2 Department of Nutrition and Dietetics, Guy’s and St Thomas’ NHS Foundation Trust, London, England, United Kingdom; 3 Department of Critical Care, Guy’s and St Thomas’ NHS Foundation Trust, London, England, United Kingdom; 4 Centre for Peri-operative Medicine and Critical Care Research, Imperial College Healthcare NHS Trust, London, England, United Kingdom; 5 Statistical Support Service, Population, Policy and Practice Programme, Institute of Child Health, University College, London, United Kingdom; 6 Institute of Health and Community, University of Plymouth, Devon, England, United Kingdom; Hospital Universitari Bellvitge, SPAIN

## Abstract

**Background:**

Muscle wasting in the critically ill is up to 2% per day and delays patient recovery and rehabilitation. It is linked to inflammation, organ failure and severity of illness. The aims of this study were to understand the relationship between muscle depth loss, and nutritional and inflammatory markers during prolonged critical illness. Secondly, to identify when during critical illness catabolism might decrease, such that targeted nutritional strategies may logically be initiated.

**Methods:**

This study was conducted in adult intensive care units in two large teaching hospitals. Patients anticipated to be ventilated for >48 hours were included. Serum C-reactive protein (mg/L), urinary urea (mmol/24h), 3-methylhistidine (μmol/24h) and nitrogen balance (g/24h) were measured on days 1, 3, 7 and 14 of the study. Muscle depth (cm) on ultrasound were measured on the same days over the bicep (bicep and brachialis muscle), forearm (flexor compartment of muscle) and thigh (rectus femoris and vastus intermedius).

**Results:**

Seventy-eight critically ill patients were included with mean age of 59 years (SD: 16) and median Intensive care unit (ICU) length of stay of 10 days (IQR: 6–16). Starting muscle depth, 8.5cm (SD: 3.2) to end muscle depth, 6.8cm (SD: 2.2) were on average significantly different over 14 days, with mean difference -1.67cm (95%CI: -2.3 to -1cm), p<0.0001. Protein breakdown and inflammation continued over 14 days of the study.

**Conclusion:**

Our patients demonstrated a continuous muscle depth loss and negative nitrogen balance over the 14 days of the study. Catabolism remained dominant throughout the study period. No obvious ‘nutritional tipping point” to identify anabolism or recovery could be identified in our cohort. Our ICU patient cohort is one with a moderately prolonged stay. This group showed little consistency in data, reflecting the individuality of both disease and response. The data are consistent with a conclusion that a time based assumption of a tipping point does not exist.

**Trial registration:**

International Standard Randomised Controlled Trial Number: ISRCTN79066838. Registration 25 July 2012.

## Introduction

A problem common to virtually all intensive care unit (ICU) patients is the deterioration in nutritional and functional status during and after their ICU stay, which has been identified as a research priority [1, 2]. In survivors of the acute respiratory distress syndrome (ARDS) muscle weakness and fatigue determined long-term outcome, it was shown that recovery time should be measured in months to years rather than days to weeks [3]. Muscle wasting and weakness were still evident one year after ICU discharge in this cohort [3], whilst physical disability had been identified up to five years later, with exercise capacity only reaching 76% of the predicted value [4]. In a similar cohort of ARDS survivors weight and fat mass increased 1 year after ICU admission, whilst lean mass losses had not yet recovered [5].

Muscle wasting during critical illness is a multi-factorial process thought to be a consequence of sepsis and inflammation, disuse atrophy, severity of illness [6,7] and hypoxia *per se* [8]. Muscle wasting may also be associated with increased cytokine levels [9, 10] and glucocorticoid administration [11]. Muscle protein breakdown rates remained high over the first week of critical illness, whilst muscle protein synthesis appeared to return to the level of a healthy fasted controls after seven days [7, 12].

Muscle mass declines early and rapidly, up to 2% per day during ICU stay [5, 7,13] with significantly higher rates of wasting seen in patients with multi-organ failure than those presenting in single organ failure [7]. For the elderly, who are increasingly represented in the intensive care population, these losses may be in addition to age-related losses and will seriously compromise recovery to independence. Low skeletal muscle mass and poor muscle quality on admission to the ICU has been shown to be a risk factor for mortality [14, 15]. Simply delivering more nutrients to critically ill patients does not appear to ameliorate muscle wasting [7, 11].

A recent research agenda review paper highlighted the need to distinguish between the different phases of critical illness (catabolic vs. anabolic phase) to help identify patients’ “readiness for enhanced feeding” [16]. Targeting nutritional strategies in the very acute catabolic phase may be counter-productive as over-feeding may be harmful [17]. Targeting interventions once peak catabolism has started to wane could therefore potentially support patients into a recovery phase without doing any harm. The aims of this exploratory research were two-fold:

1. To explore the relationship between muscle depth loss (measured via ultrasound) and nutritional and inflammatory markers during prolonged critical illness using standard bedside equipment.
2. To identify when during prolonged critical illness catabolism might decrease such that targeted nutritional or pharmacological strategies could be initiated.

## Materials and methods

The study received a favourable ethical review from Camden & Islington Research Ethics Committee (10/H0722/40), which included approval of the consent procedure for patients lacking capacity. This study was conducted in four adult intensive care units (mixed medical, surgical and trauma patients) in two large London teaching hospitals from September 2010 to February 2013. All patients anticipated to be ventilated for >48 hours were considered for inclusion into this study. Participants were excluded if they were permanently wheelchair bound; were not able to provide retrospective consent due to learning difficulties, psychiatric reasons or dementia; were receiving long term steroid treatment, had severe Parkinson’s disease, had bilateral amputations or if they were pregnant.

### Descriptive measures

Serum C-reactive protein (CRP), urinary urea and 3-methylhistidine (3-MH), nitrogen balance and total muscle depth were measured serially in patients.

Muscle depth change (cm) was measured using a Sonosite M Turbo^™^ ultrasound machine with a 5 MHz linear array transducer (Sonosite Ltd, Hitchin, Hertfordshire, UK). Muscle depth change was assessed over the bicep, forearm and thigh following the protocol described by Reid *et al* [13]. Reid provided training to the investigator using the following protocol:

Participants were supine with measurements made on the right side of the body, on the bicep, forearm and thigh to mark a halfway point on the limb from which the ultrasound measurement would be made. Markings were made in indelible ink to ensure that the same site would be measured throughout the patient’s ICU stay. Three ultrasound measurements were made per site using the built-in electronic calliper on a frozen real-time cross-sectional image. The average of three measurements for each site was used, up to a 0.2cm difference was accepted. The mean value from the bicep, forearm and thigh was combined to provide a daily total muscle depth (cm). A substantial amount of ultrasound gel was applied to ensure that the probe could rest gently on the skin without compressing muscle or distorting underlying soft tissue.

#### Bicep

The elbow was flexed to 90degress and a point on the skin was marked between the tip of the olecranon and the acromion with indelible ink. With the elbow extended and the patient in a supine position, the forearm was supinated. The ultrasound probe was applied at the pen mark on the upper arm to obtain a cross-sectional (axial) view which included the humerus, biceps and brachialis muscle, subcutaneous tissue and skin.

#### Forearm

The patient’s arm was extended and forearm kept in supinated position. A point between the antecubital skin crease and the ulnar styloid was marked with indelible pen. The ultrasound probe was applied at the pen mark on the radial (lateral) side of the forearm to obtain a cross-sectional (axial) view which included the radius. The thickness of the flexor compartment was measured anteriorly between the superficial fat-muscle interface and the interosseous membrane; radial or lateral side of the forearm.

#### Thigh

The patient was supine with knee extended. A halfway point on the thigh was identified and marked. The thickness of the quadriceps muscle group anteriorly between the superficial fat-muscle interface and the femur was measured (Vastus intermedius and Rectus Femoris). The ultrasound probe was applied at the pen mark on the anterior surface of the thigh to obtain a cross-sectional (axial) view which included the femur, quadriceps muscles, subcutaneous tissue and skin.

Reliability tests were performed between investigators prior to conducting this study, intra-class correlation coefficients were 0.984, 95%CI (0.958–0.993) and 0.965, 95%CI (0.882–0.990) respectively for intra- and inter-rater assessments, indicating good reliability. Bland Altman data for intra-rater assessment: mean difference -0.05cm, 95% upper limit: 0.73cm, 95% lower limit: -0.83cm. Bland Altman data for inter-rater assessment: mean difference 0.02cm, 95% upper limit: 1.04cm, 95% lower limit -1.00cm.

C-reactive protein (mg/L) was measured from plasma samples collected on days 1, 3, 7 and 14 of the study. Urinary studies from study days 1, 3, 7 and 14 were analysed in accredited clinical laboratories and included analysis of 3-methylhistidine (μmol/24h), as a surrogate marker for skeletal muscle breakdown, and urinary urea (mmol/24h). Urinary 3-methylhistidine was selected as it appears almost solely in skeletal muscle protein and is not re-used.

Urinary 3-methylhistidine were analysed using an amino acid analyser with cation exchange chromatography (JEOL UK Ltd., Hertfordshire, UK). Samples were analysed using ninhydrin detection and one inferred standard to quantify amino acid content. External quality assurance was provided by ERNDIM Quantitative Amino Acid Scheme. Urine urea was measured by a kinetic urease using an Abbott Architect assay with Abbott reagents (Abbott Diagnostics, Berkshire, UK). External quality assurance was provided by UKNEQAS Urine Chemistries Scheme. No reference ranges are provided for urinary urea or 3-MH excretion per 24h.

Nitrogen balance (g/day) was calculated using both the British Dietetic Association’s Parenteral and Enteral Nutrition Group [18] recommended equation and the Deacon equation [19] for estimating nitrogen excretion, assuming nitrogen balance equals nitrogen intake minus nitrogen excretion.

#### Nitrogen balance equations

The British Dietetic Association’s Parenteral and Enteral Nutrition Group (PENG) nitrogen balance equation [18]:

urinary urea (mmol/24h) x 0.033 + obligatory losses (2-4g nitrogen/24h for hair, skin and faecal losses). Add 0.6g nitrogen per 1 degree above 37.5°C.

The Deacon equation for nitrogen balance:

urea excretion (mmol/24h) x 0.028. Add 20% for other urinary losses and a further 2g/day for losses by other routes [19].

Weight (kg), height (m) and body mass index (kg/m^2^) data were recorded in addition to daily energy (kcal) and protein (grams) intakes. Recent weights and heights were obtained from medical notes. If this was not available family members were asked or weight was estimated by an experienced dietitian. If height data was not available heights were estimated from obtaining ulnar length (measured from the acromion to the ulnar styloid). Enteral feed tolerance was monitored by assessing gastric residual volumes (ml) and incidence of vomiting. Patients were fed within 48h of ICU admission according to the local ICU feeding protocol; energy requirements were calculated with predictive equations [20, 21] and Propofol derived energy was included. No indirect calorimetry was available. Protein requirements were estimated at 1.2–1.5g/kg/day [21, 22].

### Statistical analysis

Continuous data were tested for normality and presented as mean (SD), mean (95% confidence intervals) or median (IQR). Categorical data are presented as frequencies. Correlations were performed using the Spearman’s rank correlation test for non-parametric data. The difference between starting muscle depth to end muscle depth was calculated by paired t-test. Descriptive statistical analyses were performed using GraphPad Prism 6.02 for Windows (GraphPad Software, La Jolla, CA). For missing data a ‘last observation carried forward’ approach was followed. A comparison of data over the first vs. the second week of study was also performed using the Wilcoxon matched-pairs signed rank test, GraphPad Prism 6.02 for Windows (GraphPad Software, La Jolla, CA). Significance was set at 0.05.

## Results

**Fig 1:**
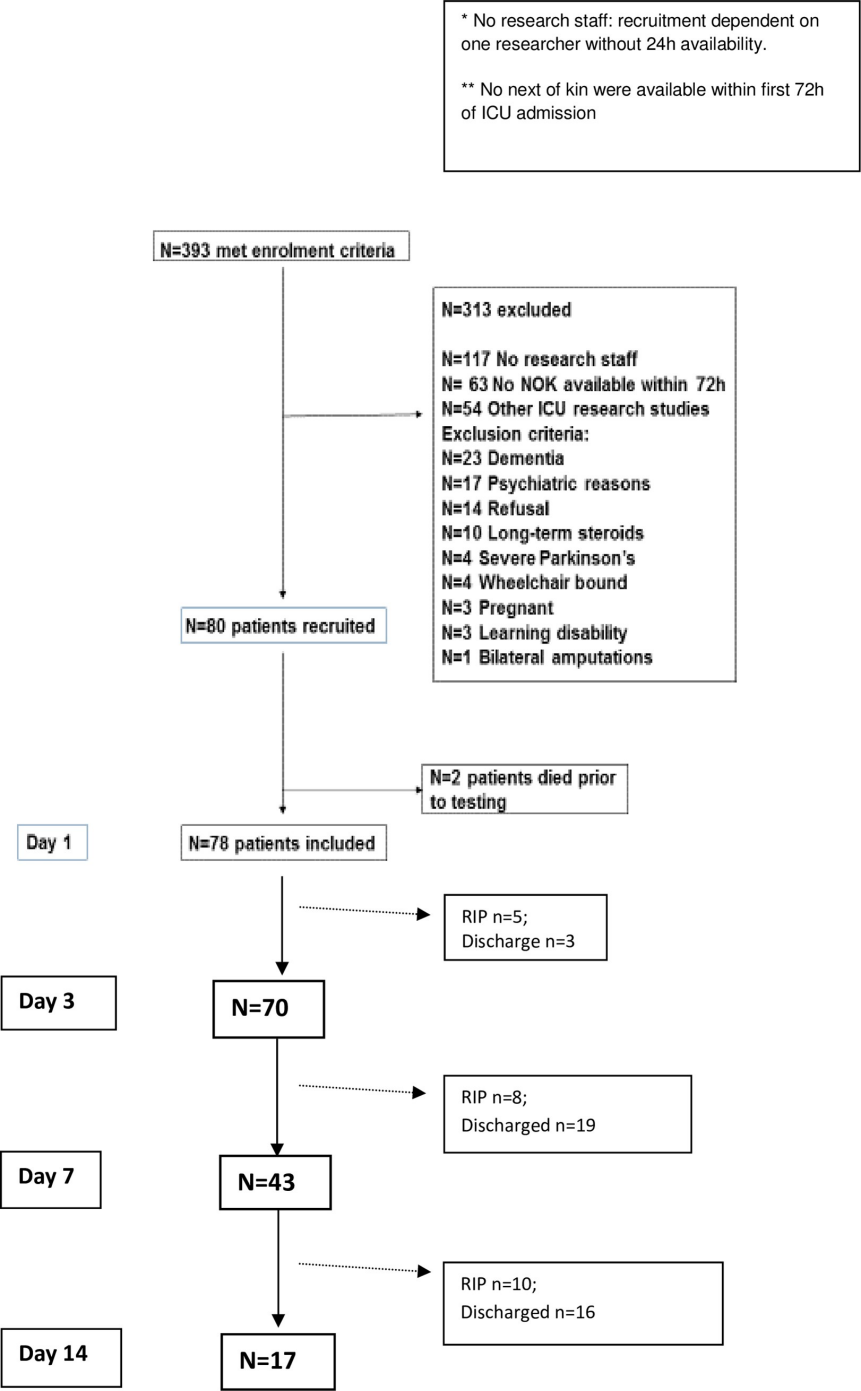
Patient recruitment on ICU.

Eighty patients were recruited to the study, two died prior to testing, 78 were included in the final analysis, **Fig 1**. ‘Other’ exclusions (**Fig 1**) refer to patients unable to provide retrospective consent due to learning difficulties or dementia, patients enrolled into other studies, patients receiving long-term steroids and previously wheelchair bound patients.

The 78 patients were representative of longer- staying patients in mixed medical and surgical intensive care units (**Table 1**) with mean (SD) age of 59 (16) years, predominantly male (69%), with a mean (SD) APACHE II score of 21.6 (7.7) and median (IQR) ICU length of stay of 10 (6–16) days. Patients were recruited within 72h of their ICU admission; ‘Day 1’ refers to day 1 of study, which occurred between days 1–3 of the patient’s ICU admission. APACHE II score was determined according to day of admission to the ICU, not day 1 of study.

**Table 1:** Patient demographics (N = 78).

Demographic factors	ICU patients N = 78
Age (years) Mean (SD) Range	59 (16) 24–89
Sex Male n (%)	54 (69%)
BMI (kg/m^2^) Median (IQR)	26 (22–31)
APACHE II score Mean (SD)	22 (7.7)
ICU Length of stay (days) Median (IQR)	10 (6–16)
Diagnostic categories—ICU admission:	
Pneumonia	19 (24%)
Cardiology/Cardiac surgery	14 (18%)
Neurology/neurosurgery	9 (11%)
Sepsis/Septic Shock	8 (10%)
Vascular surgery	6 (7.6%)
Major Trauma	6 (7.6%)
Traumatic Brain Injury	6 (7.6%)
Gastroenterology	3 (3.8%)
Gastrointestinal Surgery	3 (3.8%)
HIV	2 (2.5%)
Multi-organ failure	1 (1.3%)
Renal Failure	1 (1.3%)

ICU: Intensive Care Unit; SD: standard deviation; IQR: interquartile range; BMI: Body Mass Index; APACHE II: Acute physiological and chronic health evaluation II; HIV: Human immunodeficiency virus.

**Fig 2:**
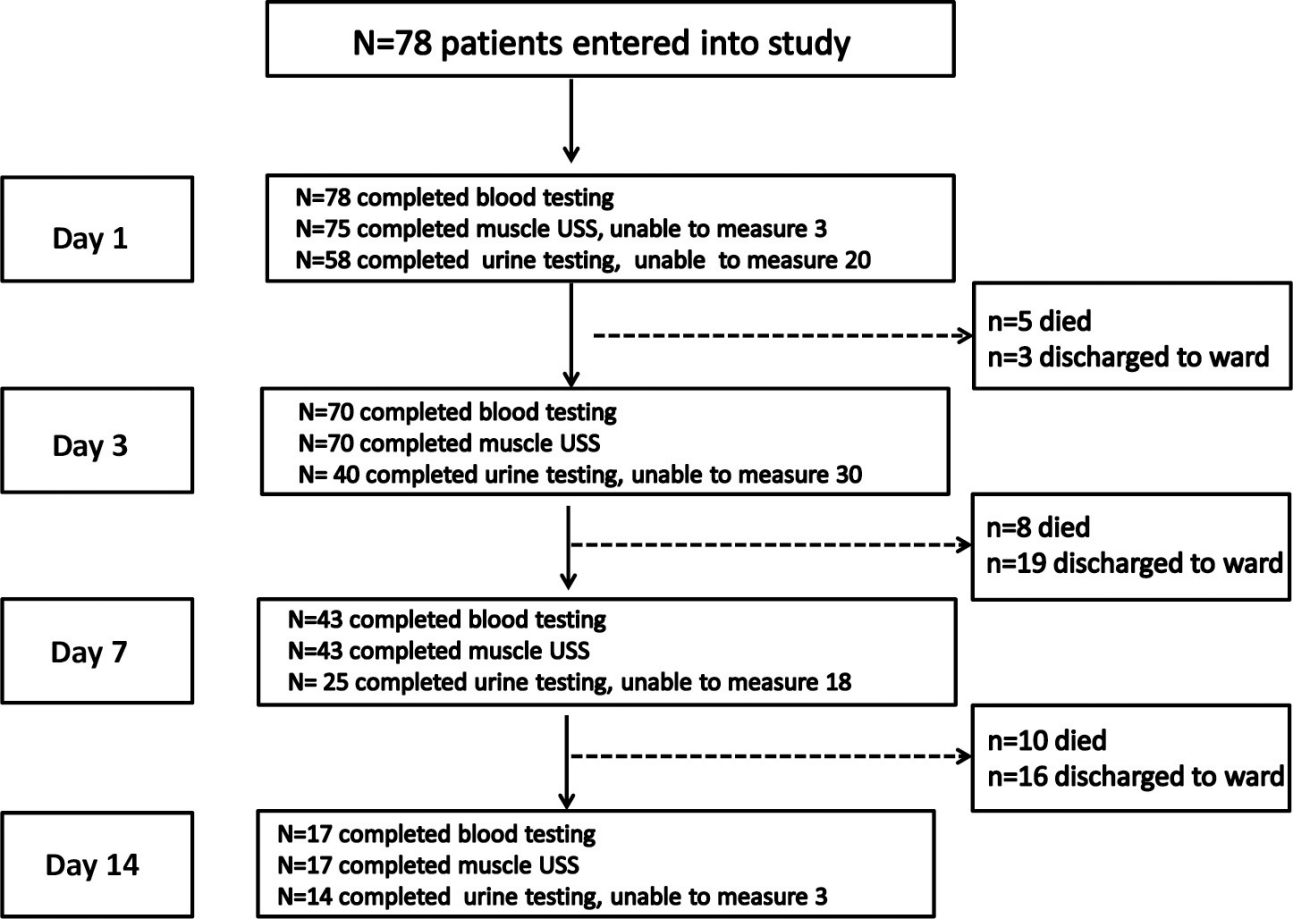
CONSORT diagram for patient discharges, deaths and patients not measured. USS: ultrasound scanning; CRRT: Continuous Renal Replacement Therapy. Blood tests: C—reactive protein and serum albumin. Urine testing not performed: day 1 (6 misplaced samples, 14 patients anuric/CRRT), day 3: (7 misplaced samples, 23 patients anuric/CRRT), day 7: (4 misplaced samples, 14 patients anuric/CRRT); day 14: (3 patients anuric/CRRT).

The flow of patients through the study is summarised in **Fig 2**, illustrating numbers achieved for each test day. Twenty-three patients (29%) died in ICU. Four patients (5%) died in hospital after ICU discharge.

There was a significant reduction in muscle depth as measured by ultrasound, over 7 and 14 days (using all available data) presented in **Table 2**. This data includes the total of three sites, the bicep (bicep and brachialis muscle), forearm (flexor compartment of muscle) and thigh (rectus femoris and vastus intermedius). **Table 3** shows the clinical characteristics of long stay patients (N = 17) studied over 14 days on ICU.

**Table 2:** Muscle depth change (total of three sites bicep, forearm, thigh).

		N = 43 over 7 days	N = 17 over 14 days
Starting muscle depth (cm)	Mean (SD)	7.6 (3.7)	8.5 (3.2)
End muscle depth (cm)	Mean (SD)	6.5 (3.1) *	6.8 (2.2) *
Mean difference (cm) P value	(95%CI)	-1.1 (-1.5 to -0.7) <0.0001	-1.67 (-2.3 to -1) <0.0001

* paired t-test

**Table 3:** Clinical characteristics of long stay patients studied for 14 days (N = 17).

	Age	Sex	Diagnosis	PMH	APACHE II score	MV (days)	ICU LOS (days)
1	72	F	Emergency AAA repair, MOF	DM II; High cholesterol, HTN	24	7	40
2	73	F	Laparotomy: ischemic small bowel, cardiac arrest. MOF	MI, PVD, IHD, Parkinson’s, COPD	17	31	51
3	59	F	Poor grade SAH for coiling, chest sepsis, MOF	Nil, previously fit and well	26	18	26
4	52	M	Pancreatitis, chest sepsis, MOF	Pancreatitis, ETOH, GORD, cholesterol, Cholecystectomy'09, smoker 10/d	17	38	57
5	80	M	VF arrest post CABG, cardiogenic shock, MOF	CHD, MI, TURP, T2DM, ESRF on HD, AF, AAA repair'97, Cholesterol	38	19	32
6	53	M	Reduced GCS, seizures, MOF	Decompensated ALD, encephalitis, Pulmonary TB, ex-smoker, previous drug taking history?	23	17	22
7	57	F	Lithium toxicity	Psychotic depression, Bell’s palsy	21	14	19
8	28	M	Myocarditis due to pneumonia	Nil	14	26	27
9	44	M	PEA arrest, chronic pancreatitis, MOF	Chronic pancreatitis, 28yr ETOH history	29	37	37
10	55	F	MSSA septicaemia	Large intra-abdominal cystic mass	9	9	17
11	85	F	Sepsis secondary to myeloma, Encephalitis	HTN, multiple myeloma—chemo	13	8	17
12	85	M	Emergency AAA repair	HTN, cholesterol, angina	16	25	25
13	24	F	Traumatic brain injury, MOF	Nil	24	17	21
14	40	M	Alcoholic Liver Disease, MOF	ALD	26	40	40
15	73	F	Chest infection	DM, HTN	14	22	24
16	77	M	Emergency AAA repair + open abdomen, MOF	HTN	27	104	108
17	32	M	Major Trauma	Nil	18	14	16

AAA: abdominal aortic aneurysm; AF: atrial fibrillation; ALD: alcoholic liver disease; CABG: coronary artery bypass graft; COPD: chronic obstructive pulmonary disease; DM: Diabetes Mellitus; ESRF: end stage renal failure; ETOH: ethanol misuse; HD: haemodialysis; HTN: hypertension; IHD: ischaemic heart disease; LOS: length of stay; MI: myocardial infarction; MOF: multi-organ failure; MSSA: Methicillin-sensitive Staphylococcus aureus; MV: mechanical ventilation; PVD: peripheral vascular disease; SAH: subarachnoid haemorrhage; TURP: transurethral resection of the prostate; VF: ventricular fibrillation

A strong association was observed between blood concentration of CRP and % muscle depth loss at day 14 (r = -0.66, p = 0.017). No correlation was observed between % muscle depth loss and age: r = -0.04, p = 0.8; APACHE II score: r = -0.124, p = 0.44; SOFA score: r = -0.14, p = 0.40 and nitrogen loss: r = -0.02, p = 0.92.

**Fig 3:**
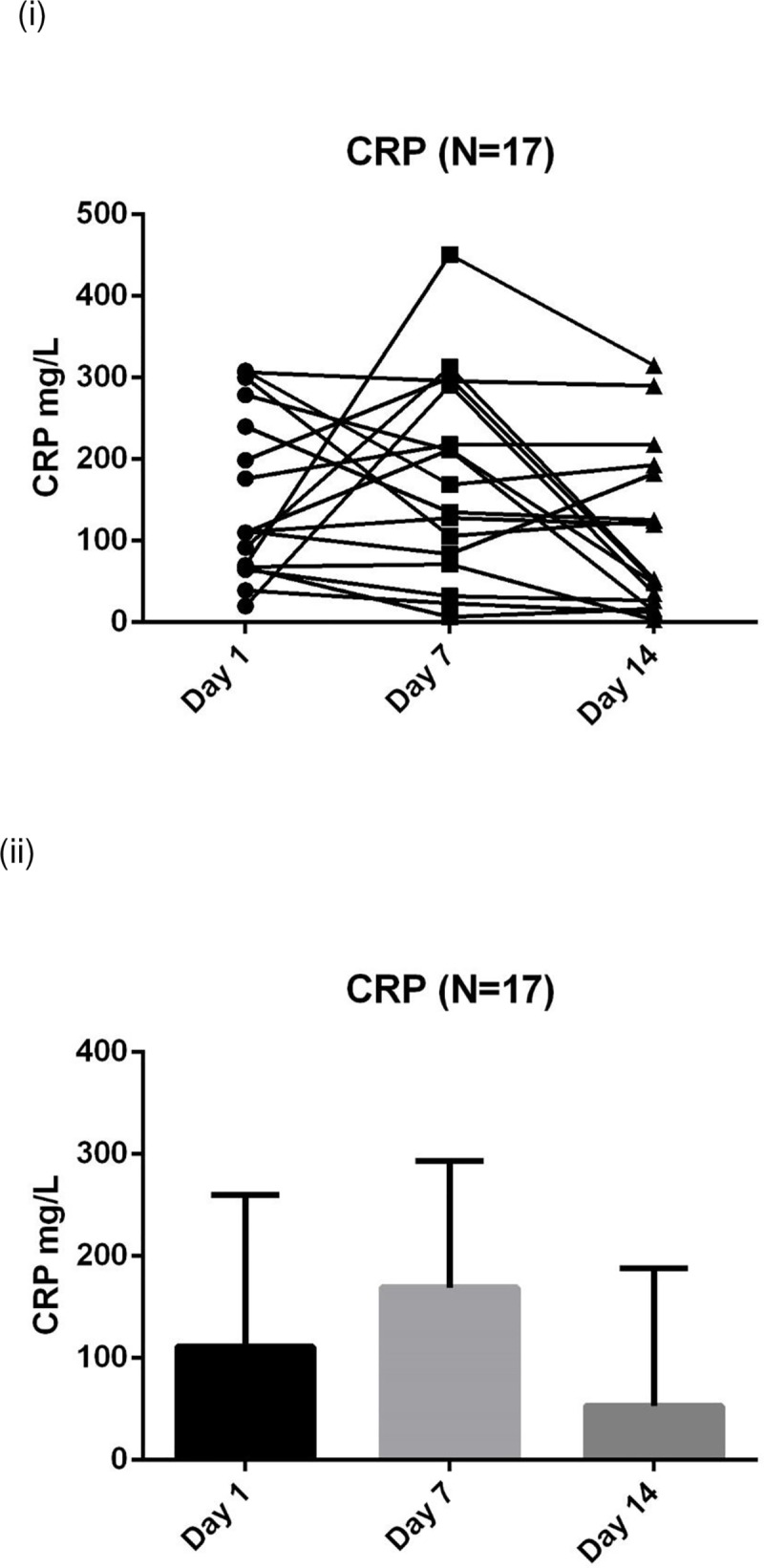
Serial CRP (N = 17) measurements in long stay patients: (i) Plots to explore individual variability (ii) Box plots reported with median and IQR. * CRP: C-reactive protein. Medians and IQR are reported in the S1 Table (a), not on this figure due to wide variance.

**Fig 4:**
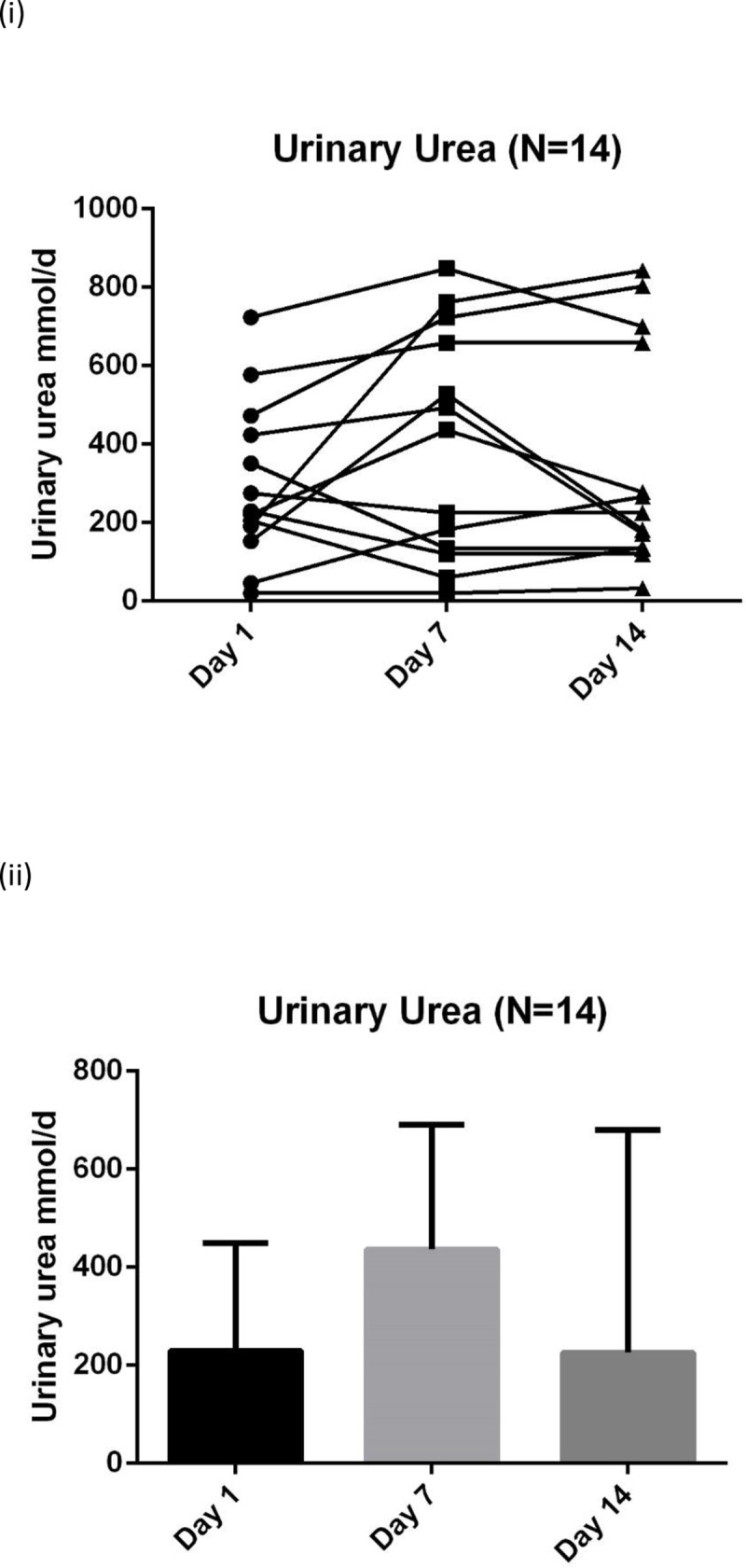
Serial urinary urea (N = 14) measurements in long stay patients: (i) Plots to explore individual variability (ii) Box plots reported with median and IQR.

**Fig 5:**
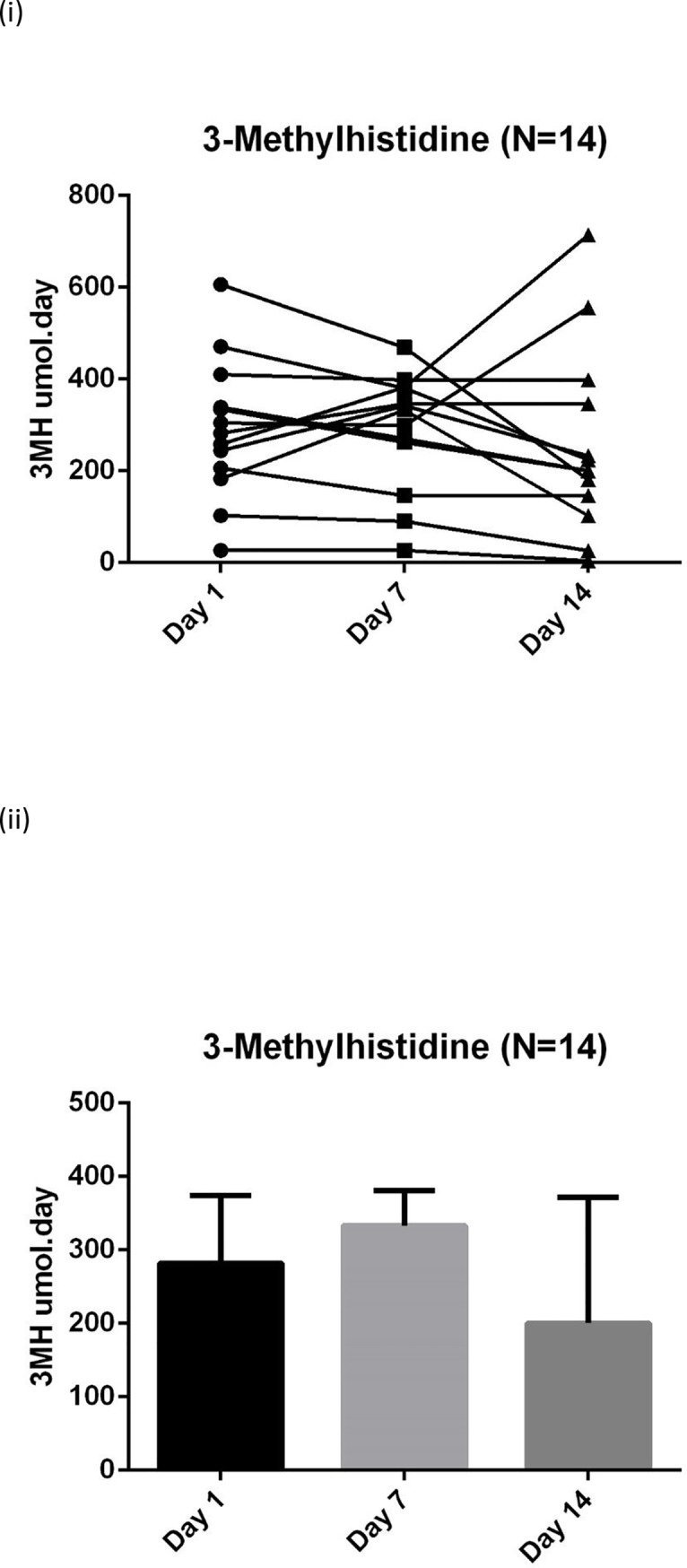
Serial 3MH (N = 14) measurements in long stay patients: (i) Plots to explore individual variability (ii) Box plots reported with median and IQR. 3-MH: 3-methylhistidine. Medians and IQR are reported in the S1 Table (a), not on this figure due to wide variance.

**Fig 6:**
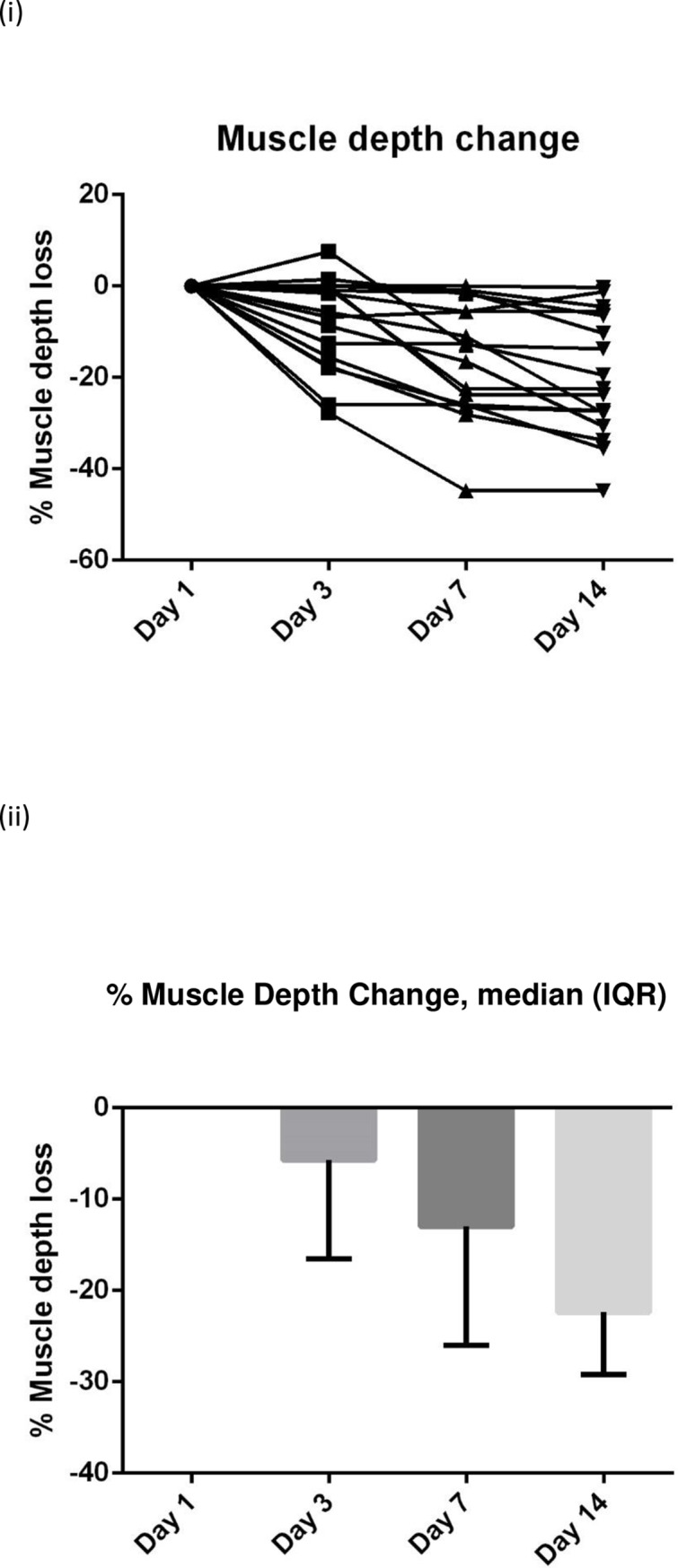
Serial % muscle depth change (N = 17) measurements in long stay patients: (i) Plots to explore individual variability (ii) Box plots reported with median and IQR.

**Fig 7:**
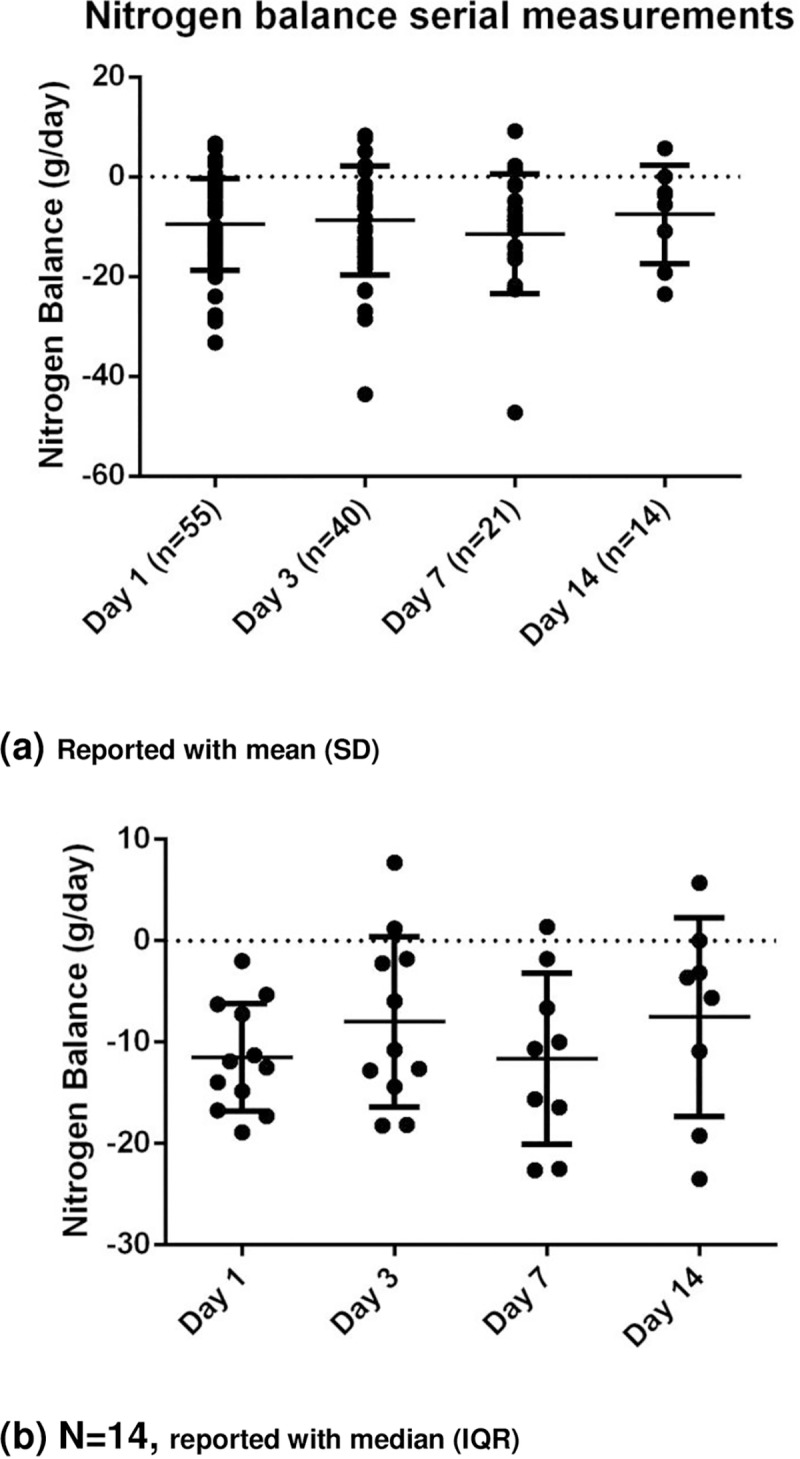
Nitrogen balance in (a) whole study cohort (N = 55) and (b) long stay patients (N = 14) over 14 days.

The biochemical data suggest that a catabolic state persists up to the end of the 14 days data collection with raised inflammatory markers, progressive protein breakdown and continuing loss of muscle depth (**Figs 4–7**). **Fig 7A** shows the negative nitrogen balance in the study cohort (N = 55) and **Fig 7B** demonstrates this negative balance in the long stay patients only (N = 14). Protein losses were included in calculations in **Fig 7A and 7B**. Visual representation of median values for urinary urea, percentage muscle depth loss, 3-MH and CRP (**Figs 3–6**) in long stay patients all suggest a persisting catabolic state up to day 14, as does the continuing negative nitrogen balance (**Fig 7A and 7B**), however of interest is the small change in trajectory of inflammatory and catabolic markers in the second week of the study data. Markers appear to improve from day 7 to 14. Median change (IQR) over 14 days is presented in a **Supporting information, S1–S3 Tables**.

In four of our patients that remained in ICU over 14 days the reduction in Day 14 CRP did not correspond with a change in surrogates for catabolism (urinary urea and 3-MH) with day 14 results still raised, **Table 4**.

**Table 4:** Cases where CRP reduced at Day 14 whilst surrogate markers for catabolism (urinary urea and 3-MH) remained high.

Patient	CRP1	CRP7	CRP14	UUrea1	UUrea7	UUrea14	3MH1	3MH7	3MH14
1	68.1	71.3	**3**	46	183	**266**	471	380	**224**
2	110	211.9	**48.2**	221.2	436.59	**277.76**	244	342	**233**
3	64.6	32.4	**27**	153	528.2	**182**	183	333	**102**
4	279.1	210.9	**13.8**	473.3	723	**802.8**	305	299	**556**

**Fig 8:**
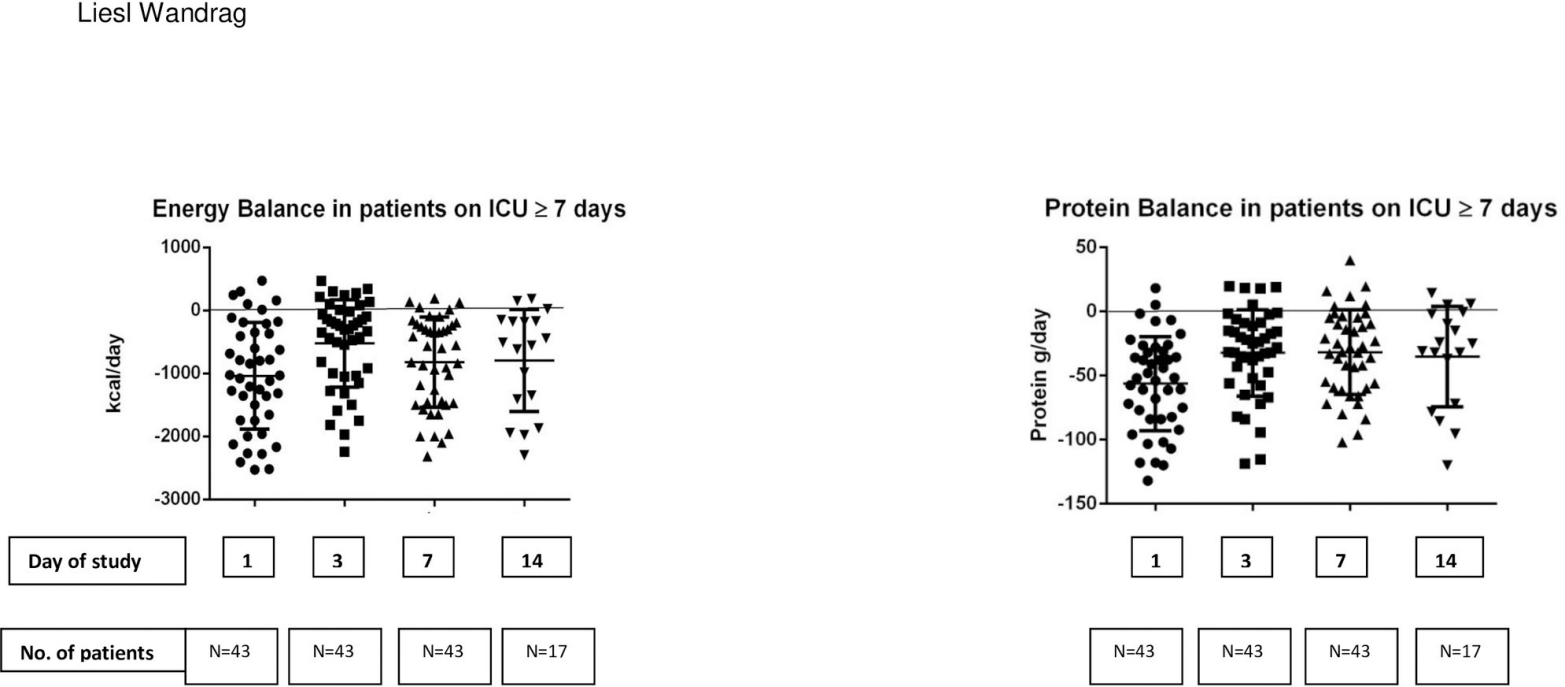
Predicted energy and protein balance of patients on the unit for ≥7 days (N = 43) over 14 days.

Energy balance (difference between estimated energy targets via predictive equations and energy intake) and protein balance (difference between estimated protein targets and protein intake) in patients remaining on the unit for ≥7 days (N = 43) is shown in **Fig 8**. Energy (and protein) targets were estimated as no indirect calorimetry was available in this study.

**Fig 9:**
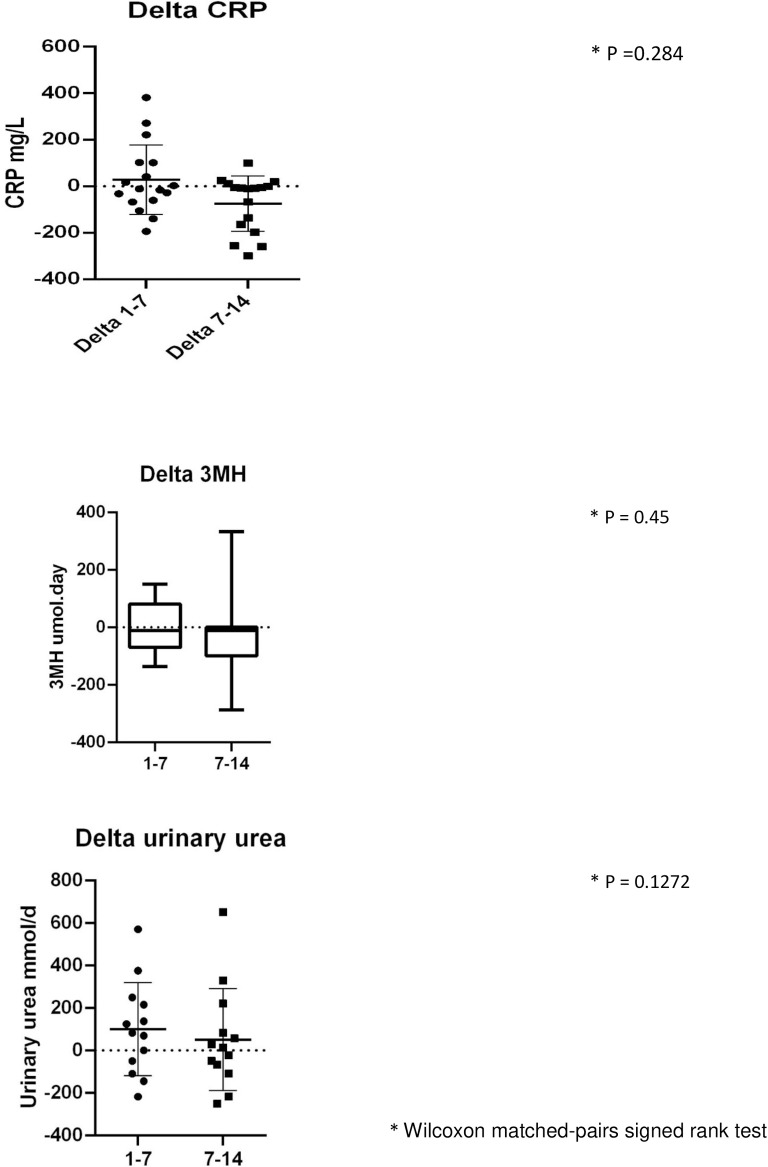
Data comparing the first versus the second week of study: Urinary urea (N = 14), 3-MH (N = 14), CRP (N = 17) and muscle loss (N = 17).

Data were also compared between the first week and the second week of study, and although they were not found to be significantly different between the two weeks, graphical representation appears to suggest a modest difference, **Fig 9**.

## Discussion

Our observational study focused on patients with a prolonged stay in ICU; the median length of stay in our study is longer than national or international figures, because we recruited only patients expected to be ventilated for >48 hours. These are patients with greater original insults or more complications that result in longer lengths of stay. This is illustrated by the day 14 data when 17 study patients still remained on ICU; 11 of these remained ventilated, two were on non-invasive ventilation, and four were self-ventilating. Although long stay patients may only represent a small group, they are an important cohort as they are resource intensive and may have outcomes that are worse than patients with an ICU stay of less than 10 days [23].

This is the first study, to our knowledge, that used clinical markers for inflammation, nutrition and catabolism in conjunction with muscle depth change measured by ultrasound to explore metabolic state during a period of critical illness. Results demonstrate progressive muscle depth loss (**Table 2**), negative nitrogen balance (**Fig 7**), continued catabolism and marginal improvement of inflammation, in patients who survive beyond 7 days (**Figs 3–6**). These results demonstrate an intense catabolic state in line with others’ work [24,25].

Our data are however highly heterogeneous with substantial individual variation. Inspection of our data does not appear to show an obvious "nutrition tipping point" within the studied cohort where anabolism or recovery could be identified, granted within a modest sample size. Additionally, the suggestion that CRP could potentially be used as a surrogate to define recovery or anabolism as inflammation subsides, has not been shown in our data. There appears to be a lack of relationship between CRP, which was used as a surrogate for inflammatory state, and markers of catabolism and muscle breakdown (24hr urinary urea and 3-MH). For some of the patients day 14 CRP results had effectively normalised yet day 14 urinary urea and 3MH were still significantly raised, indicating ongoing catabolism. This points to the need for individual monitoring using a number of markers to understand catabolism.

Although we initially aimed to identify a ‘nutritional tipping point’, which could be used to predict whether patients remained catabolic or not using logistic regression, it was difficult to define ‘catabolism’ accurately from the markers measured in this study. If nitrogen balance, urinary urea and urinary 3-methylhistidine are used as surrogates for catabolism, there is potential for missing data for patients on renal replacement therapy.

Enteral feeding should commence early in the critically ill, within 24-48h of ICU admission [21, 22], what is less clear is how much we should feed patients during the first weeks of illness. Tailoring energy targets to the metabolic phase of critical illness is widely discussed [16, 26], however determining how to identify the phases remains challenging. It has been recognised that a ‘dynamic marker’ is required to help identify which patients might be ready for increased feeding. This type of marker would ideally be able to track changes in endocrine and metabolic markers [16]. Yet there is no agreed definition on the exact description of the phases, when they might occur or how one would identify the transition point between them. ESPEN guidelines [21] recently expanded the definition beyond the ‘ebb’ and ‘flow phase’, with an early acute phase (where patients have metabolic instability and with increasing catabolism) and a late acute phase (where metabolic disturbances have started to settle down whilst significant muscle wasting is observed). Beyond 7 days has been defined as the ‘post-acute phase’ in which patients either progress onto rehabilitation, or remain in a ‘persistent inflammatory or catabolic state’, which will require a prolonged period of hospitalisation [21]. Regardless of the definition used our impression is that identification of these phases and the transition points between them will be very challenging to determine, particularly at a population level. We believe that there is need to move towards a more individualistic system, but how this can be achieved remains uncertain. Whether metabolomic profiling could be used as future way to distinguish metabolic phases [26] remains to be seen.

It is also not yet known whether catabolism could be reduced early, or at all during ICU stay. Future strategies to reduce early catabolism might require as yet unidentified pharmacological interventions or, more speculatively, nutritional interventions coupled with physical activity or a combination thereof. Timing, dose and duration of nutritional intervention are all important factors to consider in future studies. Nutritional interventions combined with physical activity would likely provide the best opportunity for recovery, since muscle anabolism is enhanced by the synergistic action of amino acids and resistance exercise [27–29]. How nutritional interventions and physical activity relate to longer term physical and functional recovery of ICU patients also needs to be explored.

Our patients lost on average 1.2% of muscle depth per day over 14 days, which confirms previous studies that report muscle depth losses between 1–2% per day [7,13]. Reid *et al* [13] reported a median 1.6% muscle depth loss per day in 48 ICU patients over 7 days, and Puthucheary *et al* [7] showed muscle losses, in patients with similar demographics to the current study, of 1.7% per day over 10 days. This group also showed that muscle breakdown remained elevated during the first week on ICU, whilst synthesis returned to normal by the end of the first week [7]. Patients in this cohort were in negative energy and protein balance (**Fig 8**), a common finding in many recent clinical trials in ICU [30–32]. Negative energy and protein balances will contribute to muscle depth loss and catabolism, however they are unlikely to be the sole cause of ICU-related muscle wasting [13,33].

Muscle ultrasound offers a practical and feasible method to serially quantify muscle mass or volume change in the ICU environment, in even the sickest patients. Ultrasound can have large inter-observer variability [22], however reliable measures are possible and have previously been reported [34]. When using this technique it is vital to ensure assessors are fully trained and intra and inter-rater reliability is tested. We carried out extensive training and found good intra- and inter-rater reliability was achieved by our assessors. A recent systematic review highlights the increased potential value and excellent reported reliability of muscle ultrasound [34].

As with all clinical studies undertaken in the intensive care environment there are a number of limitations. As previously mentioned this study cohort consisted of a group of patients with a moderately prolonged stay on ICU, which limits the wider applicability of the findings. The attrition of patients from the study cohort due to discharge from ICU to wards has meant a large amount of missing data at later time points, complicating the interpretation of the data. We were unable to follow patients up on the wards due to limited resources. We would recommend that follow up after discharge from ICU should be included in future research. Similarly mortality in the study cohort made data interpretation more difficult as some of the data presented is from patients who ultimately died. Additionally, patients on continuous renal replacement therapy were excluded from urine collection studies. Protein losses in the critically ill could be underestimated from urinary urea [35], and muscle breakdown may not be the sole contributor to 3-MH excretion as actin is present in other cells [36]. Additionally, as 3-MH is derived from actin and myosin, breakdown reflects that of all muscle which may include skeletal, smooth and cardiac muscle. Using 3-MH as a surrogate measure for muscle breakdown in conjunction with other outcome measures to detect muscle or protein breakdown may reduce this limitation. Energy expenditure was estimated using predictive equations rather than measured by indirect calorimetry as calorimetry was not available. Energy and protein balances may appear artificially low in our study as balances were calculated over a 24h period rather than accounting for part days on the ICU. Finally, day 1 of study may not necessarily be the first day of the patient’s ICU admission (median ICU stay at day 7 of study was 8 days).

## Conclusion

Strategies to limit muscle loss are much needed in this population to aid recovery, and the timing of such strategies may be crucial to their success. Our patients demonstrated a continuous muscle depth loss and negative nitrogen balance over the 14 days of the study. Catabolism remained dominant throughout the study period whilst there appeared to be a marginal improvement in inflammation. No obvious ‘nutritional tipping point” to identify anabolism or recovery could be identified in our cohort. Our ICU patient cohort is one with a moderately prolonged stay; a very important group due to their high resource requirement. This group showed little consistency in data, reflecting the individuality of both disease and response. Thus the data are consistent with a conclusion that a time based assumption of a tipping point does not exist. This supports an argument for individually characterising patient state and personalising the approach; be that with a yet to be identified metabolic marker, a novel method or a device.

### Key messages

- In this ICU patient cohort with moderately prolonged stay, catabolism remained elevated throughout 14 days follow up even though inflammation improved marginally.
- CRP does not appear to be a good surrogate marker to assess anabolism and recovery.
- Our data did not demonstrate an obvious time point where anabolism starts to exceed catabolism or ‘nutritional tipping point’.
- There is a need to individually characterise patient state and personalise the approach.

## Supporting information

S1 Table All long stay patients: Median biomarker change (IQR) over 14 days on ICU. (DOCX)

S2 Table Survivors: Median biomarker change (IQR) over 14 days on ICU. (DOCX)

S3 Table Non-survivors (ICU and hospital deaths): Median biomarker change (IQR) over 14 days on ICU. (DOCX)

## Abbreviations

APACHE II: Acute Physiology and Chronic Health Evaluation score II
ARDS: acute respiratory distress syndrome
CRP: C—reactive protein
ICU: Intensive care unit
IQR: Interquartile range
3-MH: 3-methylhistidine
PENG: parenteral and enteral nutrition group
SOFA: Sequential Organ Failure Assessment.

## References

[1] NICE 2. Rehabilitation after critical illness (CG83). 4-10-2009. Online Source.

[2] Bear DE, Wandrag L, Merriweather JL, Connolly B, Hart N, Grocott MPW and on behalf of the Enhanced Recovery After Critical Illness Programme Group (ERACIP) investigators. The role of nutritional support in the physical and functional recovery of critically ill patients: a narrative review. Critical Care (2017) 21:226 10.1186/s13054-017-1810-2 28841893 PMC6389279

[3] Herridge MS, Cheung AM, Tansey CM, Matte-Martyn A, Diaz-Granados N, Al Saidi F, et al One-year outcomes in survivors of the acute respiratory distress syndrome. N Engl J Med 2003 2 20;348(8):683–93. 10.1056/NEJMoa022450 12594312

[4] Herridge MS. The challenge of designing a post-critical illness rehabilitation intervention. Crit Care 2011 10 25;15(5):1002 10.1186/cc10362 22047913 PMC3334736

[5] Chan KS., Mourtzakis M, Friedman L, Dinglas VD, Hough CL, Wesley E, et l. Acute Respiratory Distress Syndrome (ARDS) Network. Evaluating Muscle Mass in Survivors of Acute Respiratory Distress Syndrome: A 1-Year Multicenter Longitudinal Study, Crit Care Med 2018. 10.1097/CCM.0000000000003183 PMC6051433 29727365

[6] Klaude M, Fredriksson K, Tjader I, Hammarqvist F, Ahlman B, Rooyackers O, et al Proteasome proteolytic activity in skeletal muscle is increased in patients with sepsis. Clin Sci (Lond) 2007 7;112(9):499–506. 17117920 10.1042/CS20060265

[7] Puthucheary ZA, Rawal J, McPhail M, Connolly B, Ratnayake G, Chan P, et al Acute skeletal muscle wasting in critical illness. JAMA 2013 10 16;310(15):1591–600. 10.1001/jama.2013.278481 24108501

[8] Wandrag L, Siervo M, Riley HL, Khosravi M, Fernandez BO, Leckstrom CA, et al, for the Caudwell Xtreme Everest Research Group. Does hypoxia play a role in the development of sarcopenia in humans? Mechanistic insights from the Caudwell Xtreme Everest Expedition. *Redox Biology* 13 (2017) 60–68. 10.1016/j.redox.2017.05.004 28570949 PMC5451185

[9] Frost RA, Lang CH. Skeletal muscle cytokines: regulation by pathogen-associated molecules and catabolic hormones. Curr Opin Clin Nutr Metab Care 2005 5;8(3):255–63. 15809527 10.1097/01.mco.0000165003.16578.2d

[10] Winkelman C. Inactivity and inflammation: selected cytokines as biologic mediators in muscle dysfunction during critical illness. AACN Clin Issues 2004 1;15(1):74–82. 14767366 10.1097/00044067-200401000-00006

[11] Hasselgren PO, Alamdari N, Aversa Z, Gonnella P, Smith IJ, Tizio S. Corticosteroids and muscle wasting: role of transcription factors, nuclear cofactors, and hyperacetylation. Curr Opin Clin Nutr Metab Care 2010 7;13(4):423–8. 10.1097/MCO.0b013e32833a5107 20473154 PMC2911625

[12] Gamrin-Gripenberg L, Sundström-Rehal M, Olsson D, Grip J, Wernerman J, Rooyackers O. An attenuated rate of leg muscle protein depletion and leg free amino acid efflux over time is seen in ICU long-stayers. Critical Care 2018 1 23;22(1):13 10.1186/s13054-017-1932-6 29361961 PMC5782367

[13] Reid CL, Campbell IT, Little RA. Muscle wasting and energy balance in critical illness. Clin Nutr 2004 4;23(2):273–80. 10.1016/S0261-5614(03)00129-8 15030968

[14] Weijs PJM, Looijaard WG, Beishuizen A, Girbes ARJ, Oudemans-van Straaten HM. Early high protein intake is associated with low mortality and energy overfeeding with high mortality in non-septic mechanically ventilated critically ill patients. *Critical Care* 2014 18:701 10.1186/s13054-014-0701-z 25499096 PMC4279460

[15] Looijaard WG, Dekker IM, Stapel SN, Girbes AR, Twisk JW, OUdemans-van Straaten HM, et al Skeletal muscle quality as assessed by CT-derived skeletal muscle density is associated with 6-month mortality in mechanically ventilated critically ill patients. Crit Care 2016 12 1;20(1):386 10.1186/s13054-016-1563-3 27903267 PMC5131531

[16] Arabi YM, Casaer MP, Chapman M, Heyland DK, Ichai C, Marik PE, et al The intensive care medicine research agenda in nutrition and metabolism. Intensive Care Med 2017 9;43(9):1239–1256. 10.1007/s00134-017-4711-6 28374096 PMC5569654

[17] Fraipont V, Preiser JC. Energy estimation and measurement in critically ill patients. JPEN J Parenter Enteral Nutr 2013 11;37(6):705–13. 10.1177/0148607113505868 24113283

[18] A Pocket Guide to Clinical Nutrition, 4^th^ Edition, Parenteral and Enteral Nutrition Group of the British Dietetic Association, 2011. ISBN 978-0-9529869-2-8.

[19] Deacon A, Sherwood RA, Hooper J, Association for Clinical Biochemistry (Great Britain). Calculations in laboratory science. ACB Venture Publications; 2009.

[20] Frankenfield D, Smith JS, Cooney RN. Validation of 2 approaches to predicting resting metabolic rate in critically ill patients. JPEN J Parenter Enteral Nutr 2004 Jul;28(4):259–64. 10.1177/0148607104028004259 15291408

[21] Singer P, Reintam Blaser A, Berger MM, Alhazzani W, Calder PC, Casaer M, et al ESPEN guideline on clinical nutrition in the intensive care unit, Clinical Nutrition (2018), 10.1016/j.clnu.2018.08.037. 30348463

[22] McClave SA, Taylor BE, Martindale RG, Warren MM, Johnson DR, Braunschweig C, et al; Society of Critical Care Medicine; American Society for Parenteral and Enteral Nutrition. Guidelines for the Provision and Assessment of Nutrition Support Therapy in the Adult Critically Ill Patient: Society of Critical Care Medicine (SCCM) and American Society for Parenteral and Enteral Nutrition (A.S.P.E.N.). JPEN 2016 2;40(2):159–211. 10.1177/0148607115621863 26773077

[23] Iwashyna TJ, Hodgson CL, Pilcher D, Bailey M, van Lint A, Chavan S, et al Timing of onset and burden of persistent critical illness in Australia and New Zealand: a retrospective, population-based, observational study. Lancet Respir Med 2016; 4: 566–73. 10.1016/S2213-2600(16)30098-4 27155770

[24] Liebau F, Wernerman J, van Loon LJ, Rooyackers O. Effect of initiating enteral protein feeding on whole-body protein turnover in critically ill patients. Am J Clin Nutr 2015 3;101(3):549–57. 10.3945/ajcn.114.091934 25733640

[25] Plank LD. Protein for the critically ill patient-what and when? Eur J Clin Nutr 2013 2 13. 10.1038/ejcn.2013.34 23403870

[26] Preiser JC, V Zanter ARH, Berger MM, Biolo G, Casaer MP, Doig GS, et al Metabolic and nutritional support of critically ill patients: consencus and controversies. Critical Care (2015) 19:35 10.1186/s13054-015-0737-8 25886997 PMC4310041

[27] Biolo G, Maggi SP, Williams BD, Tipton KD, Wolfe RR. Increased rates of muscle protein turnover and amino acid transport after resistance exercise in humans. Am J Physiol 1995 3;268(3 Pt 1):E514–E520. 10.1152/ajpendo.1995.268.3.E514 7900797

[28] Cermak NM, Res PT, de Groot LC, Saris WH, van Loon LJ. Protein supplementation augments the adaptive response of skeletal muscle to resistance-type exercise training: a meta-analysis. Am J Clin Nutr 2012 12;96(6):1454–64. 10.3945/ajcn.112.037556 23134885

[29] Churchward-Venne TA, Murphy CH, Longland TM, Phillips SM. Role of protein and amino acids in promoting lean mass accretion with resistance exercise and attenuating lean mass loss during energy deficit in humans. Amino Acids 2013 5 5. 10.1007/s00726-013-1506-0 23645387

[30] Harvey SE, Parrott F, Harrison DA, Bear DE, Segaran E, Beale R, et al Trial of the route of early nutritional support in critically ill adults. N Engl J Med 2014 10 30;371(18):1673–84. 10.1056/NEJMoa1409860 25271389

[31] Heyland DK, Cahill NE, Dhaliwal R, Sun X, Day AG, McClave SA. Impact of enteral feeding protocols on enteral nutrition delivery: results of a multicenter observational study. JPEN J Parenter Enteral Nutr 2010 11;34(6):675–84. 10.1177/0148607110364843 21097768

[32] Wandrag L, Gordon F, O'Flynn J, Siddiqui B, Hickson M. Identifying the factors that influence energy deficit in the adult intensive care unit: a mixed linear model analysis. J Hum Nutr Diet 2011 2 21. 10.1111/j.1365-277X.2010.01147.x 21332838

[33] Hasselgren PO. Catabolic response to stress and injury: implications for regulation. World J Surg 2000 12;24(12):1452–9. 10.1007/s002680010262 11193708

[34] Connolly B, MacBean V, Crowley C, Lunt A, Moxham J, Rafferty GF, et al Ultrasound for the assessment of peripheral skeletal muscle architecture in critical illness: a systematic review. Crit Care Med 2015 4;43(4):897–905. 10.1097/CCM.0000000000000821 25559437

[35] Dickerson RN, Tidwell AC, Minard G, Croce MA, Brown RO. Predicting total urinary nitrogen excretion from urinary urea nitrogen excretion in multiple-trauma patients receiving specialized nutritional support. Nutrition 2005 3;21(3):332–8. 10.1016/j.nut.2004.07.005 15797675

[36] Chinkes DL. Methods for measuring tissue protein breakdown rate in vivo. Curr Opin Clin Nutr Metab Care 2005 9;8(5):534–7. 16079625 10.1097/01.mco.0000170754.25372.37

