# Supplementary material for: Exploration of muscle loss and metabolic state during prolonged critical illness: Implications for intervention?
Source: PLoS One. 2019 Nov 14;14(11):e0224565. doi: 10.1371/journal.pone.0224565 (PMC6855435; doi:10.1371/journal.pone.0224565)
Supplement: S1 Table — (DOCX) [file pone.0224565.s001.docx]

**Supporting information**

**S1 Tables**: **All long stay patients**: **Median biomarker change (IQR) over 14 days on ICU**

|  | **Day 1** | **Day 3** | **Day 7** | **Day 14** |
| --- | --- | --- | --- | --- |
| Median muscle depth loss, % (IQR) | **N=17**  **0%** | **N=17**  **-1%** (-16.8 to 0) | **N=17**  **-16.5%** (-26 to -1.5) | **N=17**  **-22.4%** (-28.95 to -6) |
| Median urinary urea, mmol/24h (IQR) | **N=14**  **229.8** (172.2 – 448.7) | **N=14**  **236.3** (96.8-  568) | **N=14**  **436.6** (127.3 to 691) | **N=14**  **225.5** (135.5 – 730.9) |
| Median CRP, mg/L, (IQR) | **N=17**  **110** (66-220) | **N=17**  **134** (67-303) | **N=17**  **211** (78-296) | **N=17**  **48.2** (13-187.9) |
| Median 3-MH, µmol/24h, (IQR) | **N=14**  **282** (195-375) | **N=14**  **262** (158-325) | **N=14**  **333** (204-381) | **N=14**  **233** (124-372) |
| Median Nitrogen Balance, g/d (IQR) | **N=14**  **-10** (-14.8 to -3) | **N=14**  **-6** (-15.8 to -1.8) | **N=14**  **-9.6** (-19.1 to -3.3) | **N=14**  **-4.6** (-17.4 to -0.8) |
