# Supplementary material for: Exploration of muscle loss and metabolic state during prolonged critical illness: Implications for intervention?
Source: PLoS One. 2019 Nov 14;14(11):e0224565. doi: 10.1371/journal.pone.0224565 (PMC6855435; doi:10.1371/journal.pone.0224565)
Supplement: S2 Table — (DOCX) [file pone.0224565.s002.docx]

**Supporting information**

**S2 Table: Survivors: Median biomarker change (IQR) over 14 days on ICU**

|  | **Day 1** | **Day 3** | **Day 7** | **Day 14** |
| --- | --- | --- | --- | --- |
| Median muscle depth loss, % (IQR) | **N=15**  **0** | **N=14**  **-3.7**  (-16.2 to 0) | **N=14**  **-11.8**  (-23.3 to -1.6) | **N=12**  **-16.5**  (-27.7 to -5.0) |
| Median urinary urea, mmol/24h (IQR) | **N=11**  **275.5**  (191.7-473.3) | **N=10**  **365.9**  (130.5-573.3) | **N=8**  **510.7**  (246.4-707) | **N=8**  **224**  (145.8-671.5) |
| Median CRP, mg/L, (IQR) | **N=15**  **110**  (67.2-240.4) | **N=15**  **113.5**  (61.5-297.3) | **N=15**  **135**  (71.3-291) | **N=15**  **48**  (13.8-125.8) |
| Median 3-MH, µmol/24h, (IQR) | **N=11**  **282**  (206-339) | **N=10**  **259**  (170.3-345) | **N=9**  **342**  (222.5-381) | **N=8**  **202.5**  (45-475.3) |
| Median Nitrogen Balance, g/d (IQR) | **N=11**  **-12.5**  (-16.7 to -6.26) | **N=10**  **-11.68**  (-15.3 to -1.05) | **N=8**  **-13.12**  (-20.98 to -7.45) | **N=8**  **-4.6**  (-17.1 to -0.79) |
