# Supplementary material for: Exploration of muscle loss and metabolic state during prolonged critical illness: Implications for intervention?
Source: PLoS One. 2019 Nov 14;14(11):e0224565. doi: 10.1371/journal.pone.0224565 (PMC6855435; doi:10.1371/journal.pone.0224565)
Supplement: S3 Table — (DOCX) [file pone.0224565.s003.docx]

**Supporting information**

|  |  |  |  |  |  |
| --- | --- | --- | --- | --- | --- |

**S3 Table: Non-survivors (ICU and hospital deaths): Median biomarker change (IQR) over 14 days on ICU**

|  | **Day 1** | **Day 3** | **Day 7** | **Day 14** |
| --- | --- | --- | --- | --- |
| Median muscle depth loss, % (IQR) | **N=11**  **0** | **N=10**  **-2.9 %**  (-8.65 to 3.2) | **N=10**  **-9.3 %**  (-12.4 to -4.3) | **N=1**  -33.7% |
| Median urinary urea, mmol/24h (IQR) | **N=5**  **147**  (87.5-386.1) | **N=4**  **391.4**  (246.5-864.8) | **N=1**  **1.1**  (1.06) | - |
| Median CRP, mg/L, (IQR) | **N=11**  **77.6**  (40-307) | **N=11**  **50**  (20.9-242) | **N=11**  **61**  (12.2-109.4) | **N=1**  **290** |
| Median 3-MH, µmol/24h, (IQR) | **N=6**  **215.5**  (54.75-434.3) | **N=4**  **353.5**  (170.3-949.3) | **N=2**  **304**  (287-321) | - |
| Median Nitrogen Balance, g/d (IQR) | **N=5**  **-6.8**  (-12.9 to 2.5) | **N=4**  **-11.5**  (-25.68 to -0.4) | **N=1**  **9.2**  (9.2) | - |
